# Supplementary material for: Multi‐dataset identification of innovative feature genes and molecular mechanisms in keratoconus
Source: J Cell Mol Med. 2024 Sep 19;28(18):e70079. doi: 10.1111/jcmm.70079 (PMC11412914; doi:10.1111/jcmm.70079)
Supplement: Supplementary file 2 — Material S2. [file JCMM-28-e70079-s002.zip › 7.GSEA analysis/3.DBNDD1/KEGG_NICOTINATE_AND_NICOTINAMIDE_METABOLISM.html]

Details for gene set KEGG\_NICOTINATE\_AND\_NICOTINAMIDE\_METABOLISM[GSEA]

|  || Dataset | DBNDD1.group.cls#h\_versus\_l |
| Phenotype | group.cls#h\_versus\_l |
| Upregulated in class | h |
| GeneSet | KEGG\_NICOTINATE\_AND\_NICOTINAMIDE\_METABOLISM |
| Enrichment Score (ES) | 0.225609 |
| Normalized Enrichment Score (NES) | 0.6034718 |
| Nominal p-value | 0.89462364 |
| FDR q-value | 1.0 |
| FWER p-Value | 1.0 |
Table: GSEA Results Summary

  

Fig 1: Enrichment plot: KEGG\_NICOTINATE\_AND\_NICOTINAMIDE\_METABOLISM      
 Profile of the Running ES Score & Positions of GeneSet Members on the Rank Ordered List

  

| SYMBOL | TITLE | RANK IN GENE LIST | RANK METRIC SCORE | RUNNING ES | CORE ENRICHMENT || 1 | NT5C1B | na | 2313 | 0.281 | 0.0532 | Yes |
| 2 | NT5E | na | 3323 | 0.238 | 0.1261 | Yes |
| 3 | NNMT | na | 7098 | 0.122 | 0.0695 | Yes |
| 4 | NMNAT3 | na | 7841 | 0.107 | 0.0936 | Yes |
| 5 | NT5C2 | na | 7919 | 0.105 | 0.1364 | Yes |
| 6 | NUDT12 | na | 8679 | 0.091 | 0.1535 | Yes |
| 7 | BST1 | na | 8796 | 0.089 | 0.1882 | Yes |
| 8 | NADSYN1 | na | 9336 | 0.081 | 0.2072 | Yes |
| 9 | NADK | na | 9797 | 0.074 | 0.2256 | Yes |
| 10 | NT5C | na | 10733 | 0.062 | 0.2250 | No |
| 11 | NNT | na | 11822 | 0.049 | 0.2145 | No |
| 12 | ENPP3 | na | 12822 | 0.040 | 0.2027 | No |
| 13 | NMNAT1 | na | 14466 | 0.027 | 0.1667 | No |
| 14 | AOX1 | na | 15596 | 0.018 | 0.1421 | No |
| 15 | NMRK1 | na | 16583 | 0.012 | 0.1188 | No |
| 16 | NT5C3A | na | 19602 | -0.010 | 0.0359 | No |
| 17 | PNP | na | 22085 | -0.033 | -0.0215 | No |
| 18 | NAMPT | na | 22749 | -0.040 | -0.0234 | No |
| 19 | NMNAT2 | na | 26172 | -0.091 | -0.0831 | No |
| 20 | NT5M | na | 26979 | -0.107 | -0.0605 | No |
| 21 | ENPP1 | na | 27846 | -0.128 | -0.0310 | No |
| 22 | CD38 | na | 27970 | -0.130 | 0.0212 | No |
| 23 | NT5C1A | na | 29048 | -0.158 | 0.0578 | No |
| 24 | QPRT | na | 31672 | -0.248 | 0.0881 | No |
Table: GSEA details [plain text format]

  

Fig 2: KEGG\_NICOTINATE\_AND\_NICOTINAMIDE\_METABOLISM      
 Blue-Pink O' Gram in the Space of the Analyzed GeneSet

  

Fig 3: KEGG\_NICOTINATE\_AND\_NICOTINAMIDE\_METABOLISM: Random ES distribution      
 Gene set null distribution of ES for **KEGG\_NICOTINATE\_AND\_NICOTINAMIDE\_METABOLISM**
